# The Prevalence of Mild Cognitive Impairment in China: A Systematic Review

**DOI:** 10.14336/AD.2017.0928

**Published:** 2018-08-01

**Authors:** Jiang Xue, Jiarui Li, Jiaming Liang, Shulin Chen

**Affiliations:** ^1^Department of Psychology, Zhejiang University, Zhejiang, China; ^2^Medical School, Zhejiang University, Zhejiang, China

**Keywords:** prevalence, mild cognitive impairment, systematic review, China

## Abstract

The aim of this study was to analyze the prevalence of mild cognitive impairment (MCI) among the aging population (60 years of age and above) in China. Epidemiological investigations on MCI in online Chinese journals were identified manually using the CQVIP, CNKI, and Wanfang databases. Articles from journals published in English were identified using PubMed and Web of Science. Original studies that included prevalence surveys of MCI were selected. Forty-eight relevant studies were included in the analysis, covering 22 provinces in China. Our results showed that the pooled prevalence of MCI in the older Chinese population was 14.71% (95% confidence interval [CI], 14.50-14.92%). The prevalence was 16.72% (95% CI, 15.68-17.71%) in clinical samples vs. 14.61% (95% CI, 14.40-14.83%) in nonclinical samples (χ2=16.60, P<0.01), and 15.20% (95% CI, 14.91-15.49%) in screened samples vs. 14.16% (95% CI, 13.85-14.46%) in diagnosed samples (χ2=22.11, P<0.01). People of older age, of female sex, or living in rural areas or western China were associated with a higher prevalence of MCI. The prevalence of MCI was high in Chinese older adults, and even higher in those who were older, female, or living in rural areas or western China. Future studies are recommended to address the prevalence of MCI in the other 12 provinces of China. Furthermore, diagnostic assessments should be included in the identification of MCI.

The concept of mild cognitive impairment (MCI), which was developed by Petersen in 1999, defines an intermediate stage between normal aging and dementia. This is especially useful in the early stages of Alzheimer’s disease (AD), where individuals have a memory impairment beyond that expected for age and education yet do not have dementia [1-3]. It is important to identify this group of people not only to develop interventions that alleviate individual suffering, but also because this represents a population at increased risk of developing dementia. Many studies indicate that MCI can be regarded as a risk factor for dementia [4-8], and some even find that MCI in older patients progresses to dementia at a rate of 60-100% over 5-10 years [9].

Many population-based studies have addressed the epidemiology of MCI in different countries. The prevalence of MCI was found to be 22.2% among people aged 71 years or older in the United States [10]. The Leipzing Longitudinal Study of Aged (LEILA 75+) reported that the prevalence of MCI was 3.1% in Germany [11]. Other studies found a prevalence of MCI of 1.03% in Canada [12], 5.3% in Finland [13], and 11.1% in Sweden [14]. Further, surveys conducted by the 10/66 Dementia Research Group in some LAMICs (low- and middle-income countries) found that the prevalence of MCI varied from 0.8% in China to 4.3% in India [15].

As the world’s most populous country with the largest aging population, China faces a severe challenge with respect to the prevention, diagnosis, and treatment of dementia. A recent study found that more than nine million people suffer from dementia in China [16], which has immense financial implications for patients, caregivers, and society. However, China remains ill-prepared for the management of this disease with inadequate health care services for dementia [17-19]. In a population-based door-to-door caregiver survey, only 26.9% of patients with dementia in China report having received a diagnosis [20].

Some studies have reported the prevalence of MCI in various regions in China. For example, the prevalence was 20.1% among community residents aged 60 or older in Shanghai [21], 15.7% among adults aged 55 or older in Beijing [22], and 4.3% among adults aged 55 or older in Hainan [23]. These studies were all conducted on smaller populations, and different studies used different inclusion criteria for participants. Moreover, even the methods of defining MCI in these investigations varied. Consequently, reliable extrapolation of the prevalence of MCI in China remains difficult without a systematic review.

In the present study, we identified epidemiological investigations on MCI among the older population (aged 60 years or older) in China. Our search produced 48 relevant full text articles for systematic analysis. We sought to identify the prevalence of MCI in China as well as stratify the rate of MCI in populations with different demographic characteristics.

## METHODS

### Search strategy

Studies were identified using the terms “mild cognitive impairments,” “MCI,” and “prevalence” in the following electronic databases: CQVIP, CNKI, Wanfang database, PubMed, and Web of Science. No attempts were taken to retrieve unpublished studies. The search did not include epidemiological studies in the areas of Hong Kong, Macao, or Taiwan because data from these areas were not easily accessible. The search returned 6764 articles, 48 of which were eligible according to our study criteria and thus included in the study analysis.

### Inclusion criteria

To give the most comprehensive characterization of MCI research in China, we set the following criteria for the selected studies: (i) full text could be accessed; (ii) case collection was based on field survey carried out in China; and (iii) among studies based on the same sample, only the best article citing superior research design and study implementation was included.

### Quality of the studies

We assessed the quality of the studies using the framework suggested by the Cochrane Collaboration: (i) accuracy of the original design, (ii) representativeness of the study sample, (iii) correctness of the method of statistical analysis, and (iv) bias analysis of the study. For inclusion decisions, quality assessment was carried out independently by two reviewers. In the case of disagreement, data were reviewed and discussed between the two reviewers. The data from all included studies were clearly tabulated, and deviations were considered and identified during the Quality Assessment stage.

**Table 1:** Study characteristics.

Refs	Location	Urban/ Rural	Clinical/Nonclinical	Diagnostic criteria	Methods	Subjects No (male/female)	MCI No. (male/female)	Prevalence (%)
24	Shanghai(E)	U	N	DSM-IV	S	1516	147	9.70(8.21-11.11)

25	Sichuan(W)	U+R	N	Petersen, 1999	S&D	3910(1923/1987)	92(35/58)	2.40(1.88-2.80)
26	Guangdong(E)	U	N	self-summarized diagnostic criteria	S	410(144/266)	88(37/51)	21.46(17.49-25.23)

27	Beijing(E)	U+R	N	Petersen, 1999	S&D	1865(897/968)	217(97/120)	11.60(10.18-13.02)
28	Guangdong(E)	U+R	N	Petersen, 1999	S&D	4697(1933/2764)	257(67/190)	5.47(4.82-6.09)

29	Guizhou(W)	U+R	N	self-summarized diagnostic criteria	S	4535(1842/2577)	680(241/439)	14.99(13.96-15.98)

30	Shanxi(W)	U	N	self-summarized diagnostic criteria	S	2895(1450/1445)	220(100/120)	7.60(6.63-8.52)
31	Zhejiang(E)	U	N	the International Working Group on Mild Cognitive Impairment, 2003	S	925(376/549)	195(134/61)	21.10(18.45-23.58)

32	Xinjiang (W)	U	N	DSM-IV	S&D	1511(686/825)	148(62/86)	9.79(8.3-11.22)
33	Xinjiang (W)	U	N	DSM-IV	S&D	2986(1435/1551)	205(134/171)	10.21(5.96-7.73)

34	22 Provinces (W+E)	U+R	N	self-summarized diagnostic criteria	S	2161	571	26.42(24.56-28.19)
35	Zhejiang (E)	U	N	DSM-IV	S	1227(536/691)	107(42/65)	10.68(7.14-10.22)
36	Jiangsu (E)	U	N	Petersen, 1999	S	1773(784/989)	243(80/163)	13.71(12.1-15.22)

37	Hainan (E)	U+R	N	Petersen, 1999	S	7665(3590/4156)	326(136/190)	4.25(3.80-4.68)

38	Guangdong (E)	unclear	C	DSM-IV	S	454(314/140)	337(239/98)	74.23(70.21-78.05)
39	Zhejiang (E)	U+R	N	Petersen, 1999	S	2164(992/1172)	310(111/199)	14.33(12.85-15.73)

40	Shaanxi (W)	U	N	Petersen, 1999	S&D	264(134/130)	35(19/16)	13.26(9.17-17.14)
41	Nei Monggol (W)	U+R	N	DSM-IV	S&D	9266(4009/5257)	1782(685/1094)	19.48(18.43-19.99)

42	Zhejiang (E)	U	N	Petersen, 1999	S&D	897(434/463)	154(53/101)	17.17(14.7-19.51)
43	Jiangxi (E)	U+R	N	Petersen, 1999	S	399(185/214)	41(24/17)	10.28(7.30-13.10)

44	Guangdong (E)	U	N	Petersen, 1999	S&D	2279(1112/1167)	167(77/90)	7.33(6.26-8.34)
45	Shaanxi (W)	U	N	Chinese prevention and treatment of cognitive dysfunction, 2006	S	1583(796/787)	396	25.02(22.88-27.04)

46	Ningxia (W)	U+R	N	Chinese prevention and treatment of cognitive dysfunction, 2006	S	2168(893/1275)	457(115/342)	21.08(19.36-22.71)

47	Hunan (E)	R	N	DSM-IV	S&D	1367(678/689)	139(65/74)	10.17(8.57-11.69)
48	Shandong (E)	U	N	diagnostic criteria summarized by Qian, 2009	S&D	1226(573/653)	115(47/68)	9.38(7.75-10.93)

49	Hubei (E)	unclear	C	self-summarized diagnostic criteria	S	597(427/170)	79	13.23(10.51-15.81)

50	Xinjiang (W)	unclear	C	self-summarized diagnostic criteria	S	598(428/170)	69(38/31)	11.54(8.98-13.97)
51	Hebei (E)	U	C	Petersen, 1999	S	2532	200	7.90(6.85-8.90)

52	Shaanxi (W)	U	N	Petersen, 1999	S	796(261/535)	145(40/105)	18.22(15.53-20.76)
53	Ningxia (W)	U	N	Chinese prevention and treatment of cognitive dysfunction, 2006	S	1033(394/6390	199(49/150)	19.26(16.86-21.55)
54	Beijing (E)	U	N	Petersen, 1999	S&D	1020(374/646)	160(61/99)	15.70(13.45-17.80)
55	Tianjin (E)	U	N	DSM-IV	S	2798(1314/1664)	339(115/224)	11.38(10.91-13.26)

56	Jiangsu (E)	U	N	the International Working Group on Mild Cognitive Impairment, 2003	S	2460(1131/1239)	450(186/264)	18.29(16.76-19.74)
57	Zhejiang (E)	U	N	self-summarized diagnostic criteria	S	1211(582/629)	251(84/167)	20.70(18.44-22.89)
58	Henan (E)	U	N	Petersen, 1999	S	1755(724/1051)	245(76/169)	13.96(12.34-15.50)

59	Anhui (E)	unclear	C	Chinese prevention and treatment of cognitive dysfunction, 2006	S	679(301/378)	92(42/50)	13.55(10.98-15.99)
60	Zhejiang (E)	U	N	Petersen, 1999	S	1906(921/985)	318(143/175)	16.68(15.01-18.27)

61	Hunan (E)	U	N	DSM-IV	S&D	1764(777/987)	229(112/175)	16.27(11.41-14.47)
62	5 Provinces (W+E)	U+R	N	Petersen, 1999	S&D	10276(4379/5897)	2137	20.80(20.01-21.54)

63	Shanghai (E)	U	N	Petersen, 1999	S	4086(1430/2656)	612(178/434)	14.98(13.88-16.02)
64	Shanghai (E)	U	N	Petersen, 1999	S&D	3141(1438/1703)	601(262/339)	20.20(17.76-20.44)

65	Shanghai (E)	U	N	the International Working Group on Mild Cognitive Impairment, 2003	S	1059(489/570)	137(65/72)	12.90(10.92-14.85)
66	Shanghai (E)	U	N	self-summarized diagnostic criteria	S	842(411/431)	180(84/91)	21.40(18.61-24.01)
67	Jilin (E)	R	N	Chinese prevention and treatment of cognitive dysfunction, 2005	S&D	976(451/519)	171(73/98)	17.60(15.14-19.78)

68	Beijing (E)	U	C	DSM-IV	S	75(63/12)	48(36/12)	11.56(10.03-13.02)
69	Shandong (E)	U+R	N	Guidelines for Diagnosis and Treatment of Dementia and Cognitive Impairment in China, 2010	S	1971(738/1233)	786	39.88(37.72-41.93)

70	Nei Monggol (W)	U+R	N	the International Working Group on Mild Cognitive Impairment, 2003	S	384(200/184)	40(18/22)	10.42(7.36-13.32)

71	Xinjiang (W)	U	N	Guidelines for Diagnosis and Treatment of Dementia and Cognitive Impairment in China, 2010	S&D	804(374/430)	223(94/127)	27.74(24.64-30.67)

E = Eastern China, W = Western China, U = urban, R = rural, C = clinical, N=nonclinical, S = screening, D = diagnosis, DSM-IV, Diagnostic and Statistical Manual of Mental Disorders-IV

## RESULTS

### Basic Information of Included Studies

Fifty-two relevant studies were found online according to the search strategy. Statistical information from 48 of these studies was collected for systematic analysis because the population samples of the remaining four studies were duplicated in other studies. Table 1 shows the characteristics of these reviewed articles [24-71].

In these 48 studies, the research timeframe ranged from 2001 to 2016. A total of 22 provinces of China was covered. Two studies recruited samples from several provinces, while the other studies each recruited samples from a single province. In addition, 92% (n=44) of these included studies reported the area of study sites as urban or rural. Among them, 29 studies were conducted in urban areas, 2 studies were conducted in rural areas, and the other 13 studies were conducted in both urban and rural areas. The total number of participants was 102,906. Additionally, 13% (n=6) of included studies used clinical samples, while the majority used nonclinical samples. Further, 65% (n=31) of the studies screened individuals for MCI with no confirmation assessment, while the remaining included a clinical diagnosis of MCI. The prevalence of MCI reported in these studies ranged from 2.40% to 74.23%.

**Table 2:** Prevalence of MCI in different populations in China

Subgroup		Cases	Population	Prevalence (%)
Age			
	60-70	2820	28386	9.93(9.59-10.28)
	70-80	3805	20612	18.46(17.93-18.99)
	≥80	1629	6234	26.13(25.04-27.22)
Gender			
	male	4356	35483	12.28(11.95-12.61)
	female	6617	51107	12.95(12.66-13.23)
China			
	Eastern China	7741	57736	13.41(13.14-13.68)
	Western China	4691	32733	14.33(13.95-14.71)
Region			
	Urban	9050	65269	13.87(13.61-14.12)
	Rural	2077	14053	14.78(14.19-15.37)
Method			
	screening	8308	54657	15.20(14.91-15.49)
	screening and diagnosis	6832	48249	14.16(13.85-14.47)
Sample Source			
	clinical	825	4935	16.72(15.68-17.76)
	nonclinical	14315	97971	14.61(14.40-14.83)

MCI, mild cognitive impairment

### Prevalence of MCI in China

A total population of 102,906 older adults was investigated, and 15,140 cases of MCI were identified. The combined pooled prevalence of MCI in China was 14.71% (95% confidence interval [CI], 14.50-14.92%). The subgroup prevalence was 16.72% (95% CI, 15.68-17.71%) in clinical samples vs. 14.61% (95% CI, 14.40-14.83%) in nonclinical samples (χ2=16.60, P<0.01), and 15.20% (95% CI, 14.91-15.49%) in screened samples vs. 14.16% (95% CI, 13.85-14.46%) in diagnosed samples (χ2=22.11, P<0.01).

**Figure 1.**
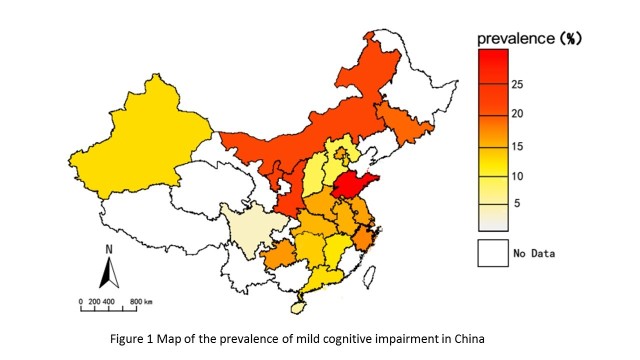
Map of the prevalence of mild cognitive impairment in China.

After excluding six studies with clinical samples and two studies in which the samples were taken from several different provinces with no specific information, 40 studies were included in analyzing the prevalence of MCI in different provinces. The prevalence of MCI in nonclinical samples varied from 2.40% in Sichuan Province to 39.88% in Shandong Province.

Figure 1 shows the map of the prevalence of MCI in different provinces. Classifying the covered provinces into eastern and western China, the prevalence was 13.41% (95% CI, 13.14-13.68%) in eastern China and 14.33% (95% CI, 13.95-14.71%) in western China (χ2=15.03, P<0.01), as shown in Table 2.

After excluding seven studies with no specific information regarding subjects’ age and fifteen studies with unmatched age divisions, 26 studies were incorporated in analyzing the aging effect on the prevalence of MCI. The prevalence of MCI increased with age, from 9.39% (95% CI, 9.59-10.28%) in those aged 60-70 years, to 18.46% (95% CI, 17.93-18.99%) in those aged 70-80 years, to 26.13% (95% CI, 25.04-27.22%) in those aged 80 years and older.

After excluding seven studies with no information on the sex of participants, 41 studies were analyzed to determine the effect of sex on MCI. There was a significant difference between sexes, with a prevalence of MCI of 12.28% (95% CI, 11.95-12.61%) in males and 12.95% (95% CI, 12.66-13.23%) in females (χ2=8.50, P<0.01). In addition, 13 studies were excluded in determining the difference between individuals living in rural and urban China. The prevalence of MCI was higher in rural areas at 14.78% (95% CI, 14.19-15.34%), compared to 13.87% (95% CI, 13.61-14.12%) in urban areas (χ2=8.01, P<0.01).

**Figure 2.**
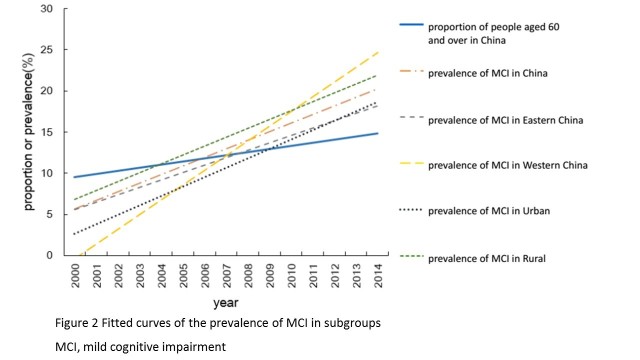
Fitted curves of the prevalence of MCI in subgroups.

Figure 2 shows fitted curves illustrating the yearly prevalence of MCI among different subgroups. The prevalence increased much more rapidly than the aging rate in China, especially in western China. There was no significant difference in the increase in prevalence between rural and urban areas.

## DISCUSSION

The main aim of our research was to analyze the prevalence of MCI in China. Fifty-two relevant studies were found, 48 of which were included encompassing 22 of the 34 provinces in China. No attempts were taken to access epidemiological studies in the areas of Hong Kong, Macao, or Taiwan because data from these areas were not easily accessible. The remaining nine provinces of China not included in the statistical analysis were Chongqing, Fujian, Gansu, Guangxi, Heilongjiang, Liaoning, Qinghai, Xizang, and Yunnan Provinces. Further relevant studies covering these provinces are recommended in the future.

A total population of 102,906 older adults was investigated in this systematic review, and 15,140 cases of MCI were identified. The pooled prevalence of MCI in China was 14.71% (95% CI, 14.50-14.92%). In other countries, the reported prevalence may be influenced by the different diagnostic criteria used, samples recruited, or assessment procedures implemented. However, the high prevalence of MCI found in this study suggests that the rate in China is much higher than that in developed countries.

Another finding of this study was the difference in the prevalence of MCI based on epidemiological factors. Specifically, MCI was higher in older vs. younger individuals, women vs. men, western vs. eastern China, and rural vs. urban areas. The aging effect has been commonly reported in previous studies [72-74], though some studies have not found a significant difference in MCI between various age groups [75, 76]. Follow-up studies would help to clarify the relationship between age and prevalence of MCI.

Women had a higher risk of developing MCI than men in this sample, which was not consistent with some studies in other countries [77]. For example, Finnish, German, and Italian studies did not find any differences based on sex [11, 78]. Peterson RC et al. (2010) reported that the prevalence of MCI was consistently higher in men than women across all ages [79]. In China, the increased prevalence of MCI in women may be the result of women having had less access to education than men in previous decades [76]. Alternatively, women may have experienced a more abrupt transition from normal cognition directly to dementia [79]. Clearly, more research is required regarding the cognitive state of elderly females in China to describe the relationship between sex and prevalence of MCI.

This study also found significant differences in the prevalence of MCI between eastern and western China and between urban and rural areas. This may be caused by different financial environments, lifestyles, and levels of access to education. Additionally, this study showed an intensely increasing rate of MCI in western China in the last decade. Indeed, while the pace of development is rapid in urban and eastern China, rural and western regions lag behind [80-82]. With increasing numbers of younger and middle-aged adults moving to developed cities for work, there is a growing demand for mental health care for older adults who remain in rural western regions. Our findings add new evidence to the need to monitor the cognitive function of older Chinese residents in rural and western regions.

Several limitations of this systematic review should be noted. First, epidemiological data on MCI in only 22 of the 34 provinces of China was included for analysis. Additionally, these studies were carried out over a period of many years, meaning that the result reported in this review was a rough estimate of the prevalence of MCI. However, since few nationwide or large-scale epidemiological investigations have been conducted, we believe that these integrated data were important in considering the epidemiology of MCI in China.

In addition, some studies were excluded in the subgroup analysis due to the lack of specific information when calculating the prevalence of MCI in different subgroups. Thus, the prevalence of MCI in the subgroups may not be based on the same population sample. Furthermore, the sample source in these studies was not unified, with a minority being clinical samples and a majority being nonclinical samples.

Finally, the methods of identifying MCI varied among the reviewed studies, such as which tool was used and whether a diagnostic assessment was performed. Different scales included the Mini Mental State Examination (MMSE), Activities of Daily Living (ADL), Montreal Cognitive Assessment (MoCA), and Clinical Dementia Rating (CDR) [83-85], making it difficult to analyze cases of MCI under a unified gold standard. Moreover, no diagnostic assessment was performed in many studies. Since MCI is a clinical stage describing a condition between normal aging and dementia, a clinical diagnosis is necessary to identify patients who are suffering from MCI [86, 87]. Simply screening would result in only a rough estimate of MCI. Future studies regarding the epidemiology of MCI should include a diagnostic assessment.

In conclusion, this study is a systematic review on the prevalence of MCI in China, which was found to be 14.71%. Individuals who were older, female, or living in rural areas or western China were associated with a higher prevalence of MCI. Increased surveillance is warranted for older Chinese residents with these characteristics for early identification and intervention before the onset of dementia. We also demonstrate the scarcity of studies on the prevalence of MCI in China. Future studies in the other provinces of China not covered in this review are recommended, and such studies should use diagnostic assessments when identifying MCI.
